# Trends in Shoulder Arthroplasty: A Narrative Review of Predominant Indications and the Most Commonly Employed Implant Designs

**DOI:** 10.3390/jcm14093186

**Published:** 2025-05-05

**Authors:** Paolo Fornaciari, Omid Jamei-Martel, Philippe Vial

**Affiliations:** 1 Clinique de La Source, Avenue Alexandre-Vinet 30, 1004 Lausanne, Switzerland; 2 Department of Orthopedics and Traumatology, University Training, Research Hospital, HFR Fribourg, 1752 Fribourg, Switzerland; omid.jamei-martel@h-fr.ch (O.J.-M.); philippe.vial@h-fr.ch (P.V.)

**Keywords:** shoulder arthroplasty, anatomic prostheses, reverse prostheses, glenohumeral osteoarthritis, proximal humerus fractures, irreparable rotator cuff tears

## Abstract

**Background:** Over the past few decades, shoulder arthroplasty has evolved rapidly, driven by a growing demand for surgical solutions to degenerative, traumatic, and irreparable rotator cuff-related pathologies, particularly in an aging but increasingly active population. **Objective:** This narrative review aims to examine the main clinical indications and the most commonly used implant designs, highlighting differences in functional outcomes, complication rates, and revision rates between anatomic total shoulder arthroplasty (ATSA) and reverse total shoulder arthroplasty (RTSA). **Methods:** Articles published between 2011 and 2025 were selected through PubMed and the Australian Joint Replacement Registry reports from 2023 and 2024. The included studies comprised randomized controlled trials, systematic reviews, and meta-analyses involving adult patients treated for primary osteoarthritis, proximal humerus fractures, and massive irreparable rotator cuff tears. **Results:** ATSA remains the preferred option in younger patients with an intact rotator cuff, due to superior outcomes in mobility and prosthesis longevity. However, glenoid component loosening remains a significant limitation. Initially reserved for irreparable cuff tears and complex fractures, RTSA has seen a progressive expansion of its indications, offering lower revision rates and satisfactory functional results, particularly in elderly patients. Recent prosthetic innovations include stemless implants, augmented glenoid components, and convertible platforms. **Conclusions:** The choice between ATSA and RTSA should be individualized, based on patient-specific factors such as age, rotator cuff integrity, functional demands, and bone quality. Advances in implant materials and design, together with improved patient selection, have significantly enhanced clinical outcomes.

## 1. Introduction

The aging population, together with improvements in quality of life, has significantly driven the search for solutions aimed at preserving individual autonomy. This is particularly relevant to the shoulder joint, which is frequently affected by significant degenerative decline.

Advancements in prosthetic design, perioperative care, and rehabilitation strategies have led to remarkable progress over the past four decades, warranting special attention.

Over time, shoulder prostheses have evolved to improve stability, durability, and biomechanical performance.

This review first examines the three conditions most commonly treated with shoulder arthroplasty: primary osteoarthritis, proximal humerus fractures, and irreparable rotator cuff tears. Subsequently, we explore the key features and clinical outcomes of the two most commonly employed implant types: anatomic total shoulder prostheses and reverse total prostheses.

## 2. Materials and Methods

The methodology for this narrative review followed the framework proposed by Grant et al., which outlines best practices for conducting narrative literature reviews [1]. Data were collected from PubMed and the 2023 and 2024 annual reports of the Australian Joint Replacement Registry to ensure inclusion of the most recent evidence on shoulder prosthetics. The selection criteria included randomized controlled trials (RCTs), case-control studies, meta-analyses, and systematic reviews involving adult patients treated for rotator cuff pathology, primary osteoarthritis, or proximal humerus fractures. Studies focusing on pathological fractures, tumors, revision for infection, or implants not covered in this review—such as shoulder interposition arthroplasty—were excluded.

In the initial phase of the literature search, the following keywords were used in PubMed: “shoulder prosthetics”, “shoulder osteoarthritis”, “proximal humerus fractures”, “rotator cuff tears”, “total shoulder arthroplasty”, “reverse shoulder prosthesis”, “clinical outcomes”, “biomechanics of shoulder implants”, “pyrocarbon shoulder prosthesis”, “anatomic total shoulder arthroplasty”, “reverse total shoulder arthroplasty”, “shoulder prosthesis outcomes”, “shoulder implant survivorship”, “shoulder hemiarthroplasty”, “rotator cuff arthropathy”, “RTSA vs. ATSA”, “glenoid loosening shoulder arthroplasty”, “pyrocarbon hemiarthroplasty shoulder”, “young patients shoulder osteoarthritis prosthesis”, and “surgical innovations shoulder replacement”. After screening titles and abstracts, full-text articles were reviewed to confirm compliance with the inclusion and exclusion criteria.

In the second phase, reference lists of the initially selected articles were also screened to identify additional studies meeting the same inclusion criteria and considered relevant for enriching the review.

Clinical outcomes and improvement in quality of life were analyzed exclusively from articles published between 2011 and 2025, selected based on their relevance to postoperative parameters such as joint function, pain reduction, and quality of life. Data synthesis was carried out using a descriptive approach, focusing on the main findings of the included studies without a formal methodological quality appraisal, consistent with the format of narrative reviews.

## 3. Discussion—Main Clinical Indications

### 3.1. Degenerative Changes in the Glenohumeral Joint—Primary Osteoarthritis

**Figure 1:**
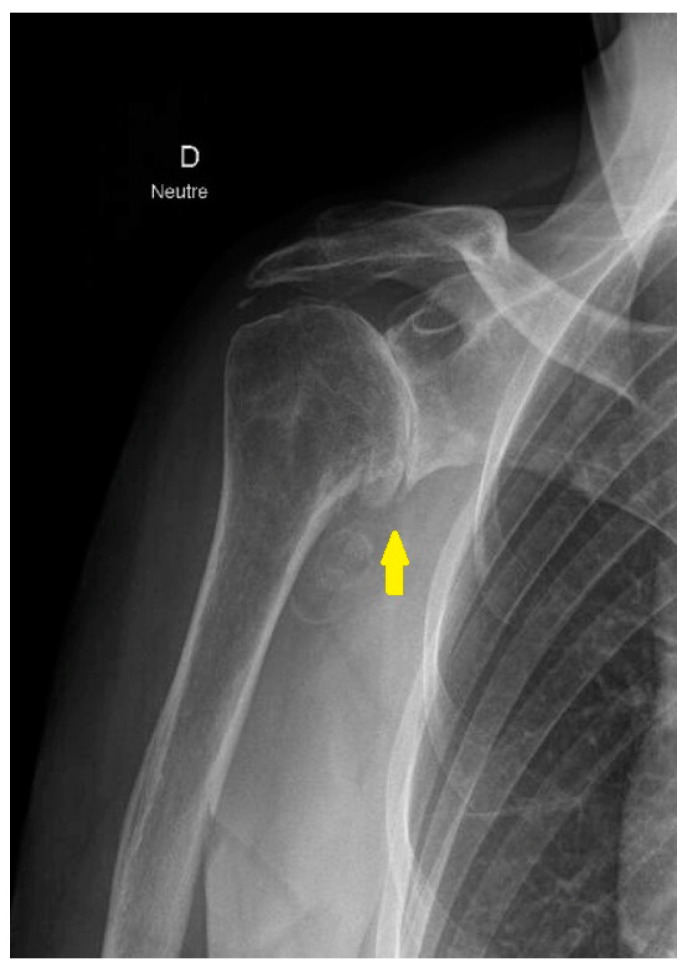
Radiograph of the anteroposterior projection of the shoulder with primary glenohumeral osteoarthritis. The yellow arrow highlights complete joint space loss, secondary to cartilage degeneration, subchondral sclerosis, and osteophyte formation—hallmarks of concentric osteoarthritis.

Degenerative changes in the glenohumeral joint are observed in up to 17% of patients with shoulder pain—a prevalence that has tripled over the past forty years, Figure 1 [2].

Common conservative treatments include physiotherapy, NSAIDs, and intra-articular corticosteroid or hyaluronic acid injections. Regenerative medicine approaches, including PRP and mesenchymal stem cell (MSC) therapy, have shown promise in managing osteoarthritis, with symptom relief lasting up to 12 months. However, limited follow-up durations, heterogeneous protocols, and restricted accessibility remain key challenges [3].

When conservative management fails to provide adequate symptom control, implantation of either an anatomic total shoulder prosthesis (ATSA) or a reverse total shoulder prosthesis (RTSA) may be considered.

Anatomic total shoulder prosthesis is widely regarded as the gold standard for treating primary glenohumeral osteoarthritis in patients with an intact rotator cuff. Long-term studies have consistently demonstrated significant improvements in pain relief and joint function [4].

Roberson et al. conducted a systematic review on patients under 65 years of age who underwent anatomic shoulder arthroplasty, reporting a mean improvement in the Constant score from 29.2 to 60.4 points, with an increase in abduction from 90° to 130° and an increase in external rotation from 15° to 39°. Implant survival rates range from 60% to 80% at 10 to 20 years of follow-up, with a mean revision rate of 17.4%. The primary cause of revision is glenoid component loosening, with radiolucent lines observed in 54% of cases [5]. Neyton et al. analyzed 202 patients under 60 years of age who underwent anatomic shoulder arthroplasty, reporting a 16.3% revision rate at 10 years, with glenoid failure accounting for 88% of these cases. Implant survival rates were reported at 95% at 5 years, 83% at 10 years, and 60% at 20 years [6].

A comparison with RTSA highlights significant differences in indications, functional outcomes, and implant longevity. Daher et al. conducted a meta-analysis of 14 studies including over 1400 patients, revealing that reverse arthroplasty was associated with a lower complication rate and a reduced reoperation rate compared to ATSA. However, patients treated with reverse arthroplasty showed a significant reduction in external rotation, while other range-of-motion parameters did not differ significantly [7].

In recent years, pyrocarbon hemiarthroplasty has emerged as a potential alternative for young, active patients with shoulder osteoarthritis. Pyrocarbon implants, characterized by their high wear resistance and an elastic modulus similar to cortical bone, aim to reduce glenoid wear compared to traditional metal implants. In a systematic review and meta-analysis by Park et al., the authors evaluated the clinical outcomes of pyrocarbon hemiarthroplasty in the shoulder, reporting significant improvements in range of motion and patient-reported outcomes. The study authors found a mean postoperative Constant score of 70.9 points, with a mean improvement of 36.2 points. However, radiographic assessments raised concerns about glenoid erosion, observed in 22.8% of patients at a minimum follow-up of two years. The overall complication rate was 8.6%, with a revision rate of 7.7%, and 63% of the revisions involved conversion to reverse or total shoulder arthroplasty. While these results are encouraging, the study underscores the need for long-term follow-up to fully assess the efficacy and durability of pyrocarbon implants in young patients with shoulder osteoarthritis [8].

Further highlighting the potential of pyrocarbon implants, McBride et al. analyzed data from the Australian Orthopaedic Association National Joint Replacement Registry, comparing pyrocarbon humeral resurfacing with metal hemi-resurfacing and metal stemmed hemiarthroplasty in patients under 55 years of age. Their findings showed that pyrocarbon hemiarthroplasty had a significantly lower revision rate at mid-term follow-up compared to other hemi-resurfacing procedures. At six years, the cumulative revision rate was 8.9% for pyrocarbon hemi-resurfacing, compared to 17.1% for metal hemi-resurfacing and 17.5% for metal stemmed hemiarthroplasty. Notably, no cases of pyrocarbon hemi-resurfacing required revision due to glenoid erosion, suggesting a potential advantage in preserving the native glenoid. Instead, the primary reasons for revision were pain, prosthesis fracture, and infection [9].

### 3.2. Proximal Humerus Fractures

Proximal humerus fractures present a significant challenge in orthopedics, particularly due to the increasing average age of the population. These injuries account for approximately 6% of all adult fractures and are more commonly observed in older individuals. Major risk factors include conditions such as osteoporosis, diabetes, and epilepsy, as well as female gender. Globally, it is estimated that around 700,000 proximal humerus fractures occur each year. This incidence is particularly high among individuals over the age of 80, with approximately 300 cases per 100,000 people annually [10,11].

The optimal management of proximal humeral fractures remains a subject of debate [12]. Displaced fractures generally require surgical intervention, whereas minimally displaced fractures are often managed conservatively [13]. Conservative treatment involves immobilization, followed by radiographic evaluation and early rehabilitation [14]. The PROFHER study found no significant long-term difference between surgical and non-surgical treatments for most fractures. However, surgery was required in 21% of cases, highlighting the need for more selective surgical indications [15,16,17]. The risk of non-union persists with conservative treatment and is influenced by factors such as head-shaft angle and smoking status [18].

**Figure 2:**
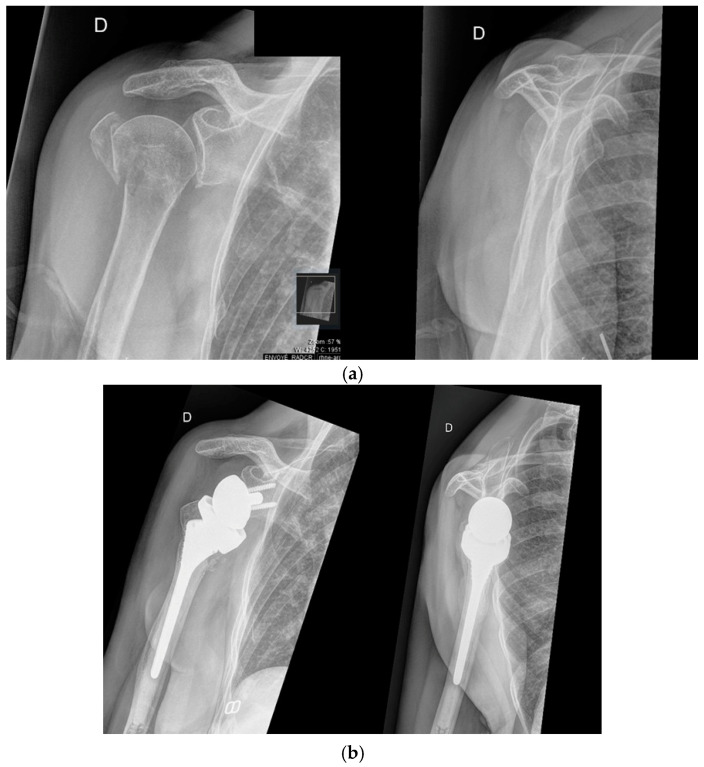
(**a**) In the figure, two radiographic projections: on the left, the anteroposterior projection and on the right, the Neer projection of a three-part fracture of the proximal humerus. (**b**) Treatment with reverse shoulder prosthesis of the fracture shown in figure (**a**), with anteroposterior projection on the left and Neer projection on the right.

Arthroplasty is typically reserved for displaced three- or four-part fractures and is generally indicated in elderly patients, with certain exceptions [13]. The two main surgical options are RTSA, Figure 2, and hemiarthroplasty (HA) [13]. In recent years, there has been a significant shift in surgical practice, with RTSA increasingly favored over HA. Reverse shoulder arthroplasty has been shown to provide better functional outcomes compared to HA, particularly in elderly patients with complex fractures. This trend is supported by evidence showing that RTSA is associated with a lower revision rate compared to HA [19,20,21,22].

Recent meta-analyses of operative treatments for proximal humeral fractures have highlighted the advantages of RTSA, demonstrating better functional outcomes compared to HA and open reduction internal fixation (ORIF), with reduced complication and reoperation rates [23]. Notably, two meta-analyses focusing exclusively on randomized controlled trials confirmed RTSA’s superiority over HA [24,25].

Fraser et al. compared RTSA and ORIF for proximal humeral fractures and demonstrated clear superiority of RTSA at two-year follow-up. RTSA achieved significantly better functional outcomes (higher Constant-Murley Score) and a lower revision surgery rate. These findings support RTSA as the preferred choice for elderly patients with complex fractures [22]. However, it is important to note that these results are based on a relatively short follow-up period of only two years [26].

Alrabaa et al. analyzed 384,158 cases, showing that RTSA use increased from 17% in 2010 to 43% in 2020, favored for elderly patients and severe fractures due to fewer complications and better outcomes. HA use dropped below 4%, and locking plate fixation (LPF) declined from 57% to 43% but remains key for younger patients despite higher complication rates. RTSA is preferred for those over 75, while LPF suits patients aged 50–70, emphasizing the need for personalized surgical approaches [27].

Cooke et al. confirmed a shift in proximal humerus fracture treatment (2010–2019). ORIF remained the most common but declined by 9%, while RTSA surged by 306%, becoming widespread across all demographics. HA was nearly abandoned, dropping by 65%. RTSA use increased by 214% in patients under 60, 244% in those 60–74, and 282% in those over 75 [28].

The timing of RTSA significantly influences outcomes: acute procedures (within 4 weeks) result in faster recovery and fewer complications compared to delayed interventions (>4 weeks), although long-term results appear similar [29].

### 3.3. Irreparable Rotator Cuff Tears and Rotator Cuff Arthropathy

**Figure 3:**
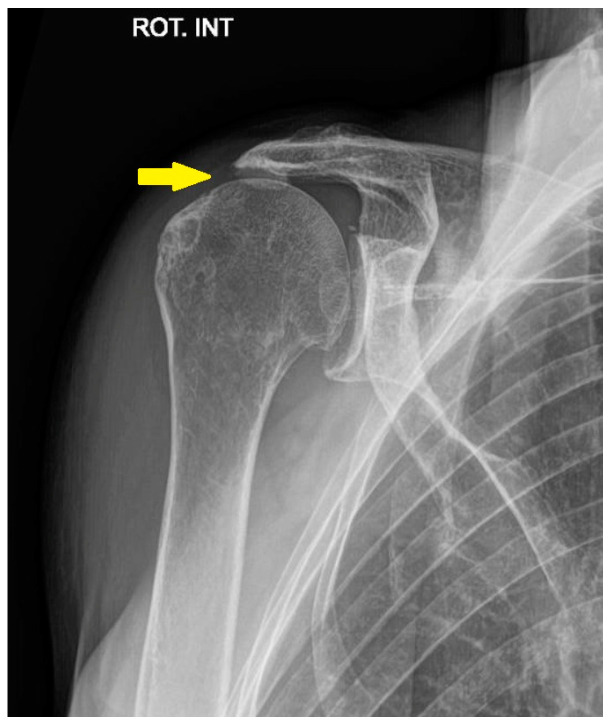
Rotator cuff arthropathy on the anteroposterior radiograph. The yellow arrow shows narrowing of the acromiohumeral space due to a posterosuperior rotator cuff tear.

**Figure 4:**
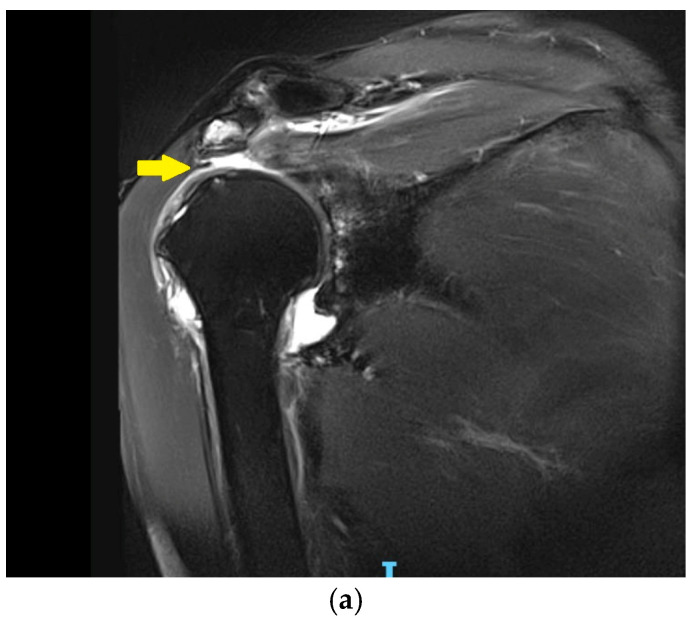

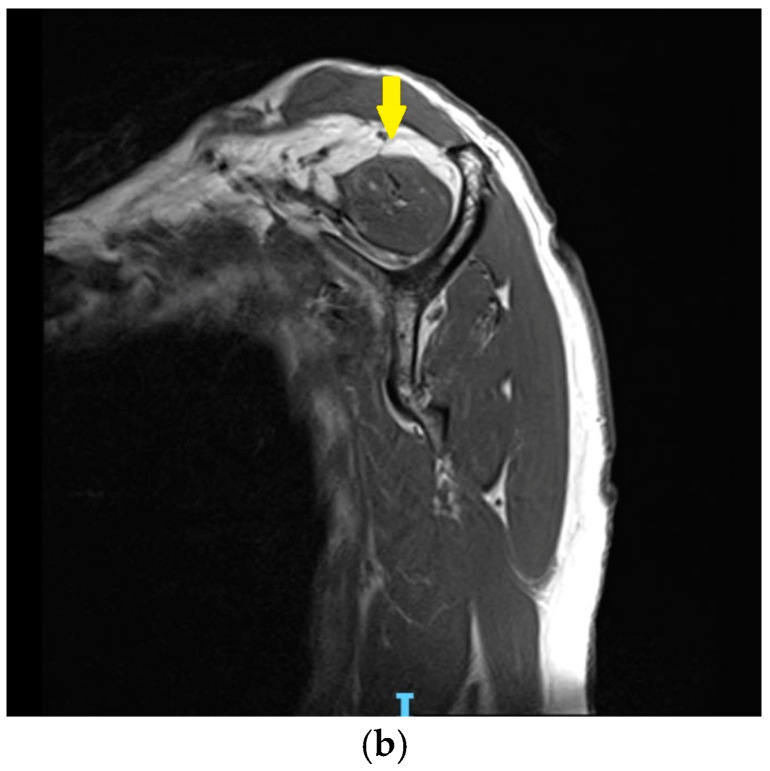
In picture (**a**), coronal T2 FS BLADE section of a right shoulder with irreparable tears of the supraspinatus and infraspinatus tendons (yellow arrow); (**b**) sagittal T1 TSE section of the right shoulder of the same patient with grade 2 muscle atrophy of the supraspinatus tendon according to Goutallier/Fuchs (yellow arrow).

The definition of irreparable rotator cuff tears may encompass cases with or without arthritis-associated glenohumeral arthritis. In general, although the definitions of irreparability vary, they typically rely on several clinical and radiological parameters. Recent classification systems have refined these definitions by incorporating tendon retraction and muscle atrophy, helping to assess reparability and guide surgical treatment options [30,31]. Irreparable rotator cuff tears present a complex orthopedic challenge (Figure 3 and Figure 4).

Management should be individualized based on tear characteristics (e.g., tendon retraction and muscle atrophy), patient age, activity level, expectations, and rehabilitation. Non-surgical treatment is usually the first choice for patients who are either unfit for surgery or prefer to avoid it [30,32]. This conservative strategy involves medications and injections (e.g., NSAIDs and corticosteroids), combined with physiotherapy to strengthen stabilizing muscles and compensate for functional deficits [30]. While this can help improve pain and function, the results are often limited, with a high failure rate of 30% [33].

Surgical options include arthroscopic repair, debridement, and biceps tenotomy; tendon transfers (e.g., latissimus dorsi and lower trapezius) for young, active patients; and superior capsular reconstruction with biological grafts to restore stability of the glenohumeral joint by preventing superior migration of the humeral head. Finally, reverse shoulder prostheses are the most effective for older patients with pseudoparalysis and irreparable tears, providing significant pain relief and functional improvements [30,32].

A recent multi-institutional study by Boin et al. on 203 patients (mean age 71 years) with massive irreparable rotator cuff tears, unresponsive to conservative treatment and treated with RTSA, showed significant improvements in shoulder mobility and function. Key outcomes demonstrated substantial postoperative improvements, with range of motion (ROM) increasing notably—particularly an average gain of 76.95° in forward elevation for patients with pseudoparalysis. The study authors reported a low complication rate of 1.6%, with three major complications, two requiring revision surgery, and a reoperation rate of 1.1%. Despite minor limitations, such as its retrospective nature and average follow-up of 50 months, these findings support RTSA as a reliable, effective treatment for lasting mobility improvements and low morbidity [34].

In recent systematic reviews and meta-analyses by Welch et al. [35] and Tantone et al. [36], the authors compared outcomes between patients with failed rotator cuff repair (RCR) and those undergoing primary RTSA, Table 1.

Welch et al. analyzed 2149 patients and found significantly worse ASES scores in the failed RCR group compared to primary RTSA. Additionally, VAS pain scores were higher in the failed RCR group. Range of motion was also reduced, with a significant decline in forward flexion, though no significant differences were noted in external rotation. Importantly, no significant differences in complication or revision rates were observed between the two groups [35].

In a study of 1590 patients, Tantone et al. confirmed these findings, reporting similar reductions in ASES scores and increased VAS pain scores in the failed RCR group. They also observed a more pronounced reduction in both forward flexion and external rotation. Moreover, the failed RCR group had a higher overall risk of complications, though revision rates remained comparable between the groups [36].

These studies highlight that patients with failed RCR experience worse functional outcomes, higher pain levels, and reduced range of motion compared to those undergoing primary RTSA. Although Welch et al. reported no significant differences in complication rates, Tantone et al. observed a higher risk of complications in the failed RCR group.

## 4. Discussion—Commonly Used Shoulder Arthroplasty Designs

### 4.1. Anatomic Total Shoulder Arthroplasty

**Figure 5:**
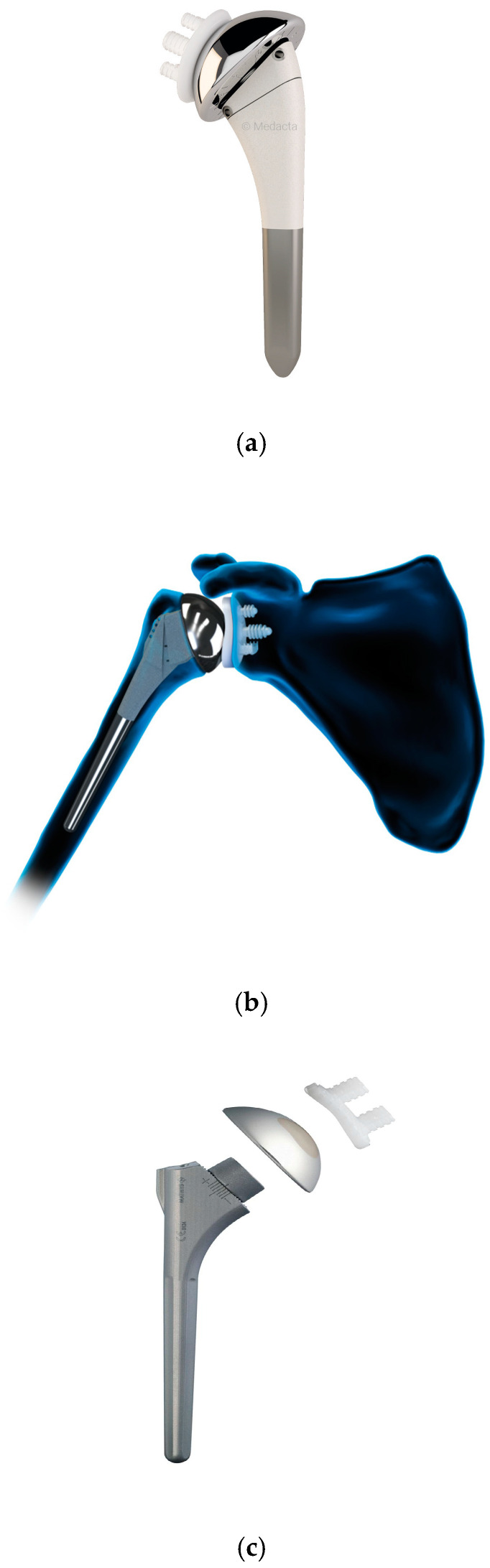
Anatomical shoulder prosthesis implants. Pictures of (**a**) Medacta, (**b**) Arthrex, and (**c**) Mathys. The implants shown in this review represent a sample of commonly used systems and do not imply endorsement of a specific manufacturer. Images reproduced with permission from the respective companies.

Anatomic total shoulder arthroplasty remains the standard treatment for advanced glenohumeral arthritis in patients with an intact rotator cuff and primary osteoarthritis, Figure 5 [4]. However, it is not suitable for those with rotator cuff tears, deltoid dysfunction, infection, Charcot arthropathy, or insufficient glenoid bone stock [37].

#### 4.1.1. Patient Factors Influencing Outcomes of Anatomic Shoulder Arthroplasty

Age plays a significant role in the survival rate of ATSA implants, which is generally lower in younger patients. For example, Australian data show a cumulative revision rate at 10 years of 16.5% in patients aged 55–64, compared to only 8% in patients over 75 years of age. In younger patients, longer life expectancy and higher physical demands place increased mechanical stress on implants, accelerating wear and loosening. In contrast, although ATSA is generally effective in older patients, complications related to rotator cuff insufficiency may arise, occasionally warranting the use of a reverse prosthesis [38].

Glenoid morphology also affects outcomes, with B2-type glenoids being particularly difficult to treat due to their marked retroversion and humeral head subluxation. Recent studies show that techniques such as eccentric reaming or the use of augmented glenoid components can yield satisfactory results [39].

Preoperative diagnosis is another key factor, as ATSA is primarily indicated for glenohumeral arthritis with an intact rotator cuff. Implant survival rates vary depending on the underlying pathology, with the best outcomes reported for rheumatoid arthritis (5.6% revision rate at 7 years) and the poorest for fracture sequelae (15.8%) [40].

#### 4.1.2. Bone and Implant Characteristics Influencing Outcomes in Anatomic Shoulder Arthroplasty

Modern prosthetic designs for the glenoid are tailored to accommodate the anatomical and pathological variations of this structure. The anatomy of the glenoid varies significantly, influencing both the choice and placement of prosthetic [41]. While the glenoid typically presents a pear-shaped form, approximately 29% of glenoids have an elliptical shape [37,41]. Glenoid implants may be cemented or non-cemented and differ in curvature radius to better adapt to the patient’s bone conditions and surgical requirements [42]. Implant selection is also influenced by glenoid retroversion and inclination, which are assessed preoperatively using various techniques to ensure optimal component positioning [43]. Neer’s cemented polyethylene glenoid components, which feature a central keel, remain a reference standard thanks to their durability and ability to accommodate anatomical variations. In osteoarthritic patients, changes in bone mineral density and morphology are critical in determining the appropriate implant, especially for managing the compressive and tensile forces acting across the joint [44]. A thorough understanding of glenoid anatomy and joint biomechanics is therefore essential to improve surgical outcomes and reduce the risk of complications such as implant loosening [37].

As for glenoid component characteristics, metal-reinforced polyethylene components—once commonly used—have shown high complication rates, including screw fractures and premature loosening [45]. This has led to the development of new-generation hybrid implants that offer better stability and extended longevity. In terms of shape, pegged designs reduce bone resection and have proven comparable to keeled designs regarding revision rates. Regarding fixation, hybrid components that combine cemented and non-cemented techniques demonstrate the best long-term outcomes [40].

The humerus also presents considerable complexity due to the three-dimensional geometry of its upper head. Early generations of humeral prostheses—monoblock or modular—could not faithfully replicate the shape or dimensions of the proximal humerus. Third- and fourth-generation implants, known as “anatomic” prostheses, not only accommodate variations in humeral dimensions thanks to their modularity but also adapt to different shapes [46]. This allows for more accurate positioning of the prosthetic head relative to the anatomical neck, which is crucial for restoring the native anatomy of the proximal humerus [47].

Regarding humeral component characteristics, selecting the appropriate humeral head size is crucial to avoid joint overload. While larger heads may improve implant survival, they can compromise range of motion if oversized [41].

#### 4.1.3. Outcomes of Total Anatomic Shoulder Arthroplasty Across Age Groups

Anatomic total shoulder arthroplasty remains an effective treatment for advanced and painful shoulder conditions. However, in patients under 60 years of age, implant longevity and high functional expectations represent notable challenges. In a recent study, researchers evaluated the mid- and long-term outcomes of 50 patients (mean age 54.1 years) who underwent ATSA, with an average follow-up of 8.7 years. The primary indications were osteoarthritis, followed by avascular necrosis and rheumatoid arthritis. Postoperative outcomes demonstrated significant improvements in shoulder function: forward elevation increased from 114° to 147° and external rotation increased from 33° to 46°. Muscle strength also improved, with both forward elevation and external rotation reaching 5/5. Pain level decreased substantially, with a final VAS score of 2.2. Functional outcomes were satisfactory, with an average ASES score of 76.4 and a Simple Shoulder Test score of 8.9. Implant survival was 98% at five years and 83.8% at 10 years. Complications occurred in 14% of patients, including glenoid loosening and infections, with revision surgery required after an average of 6.5 years. These findings support the use of ATSA as a viable option even in younger, active patients [48].

A recent study by Jensen et al. reinforces the continued role of anatomic prostheses in older, active patients with an intact rotator cuff. In a cohort of patients aged 70 years and older (mean age: 76.2) and a mean clinical follow-up of 3.3 years, clinical outcomes were excellent. The mean postoperative VAS pain score was 1.6, and 55% of patients reported being pain-free. Forward elevation improved from 96° to 160°, external rotation increased from 26° to 64°, and 71.8% of patients achieved internal rotation to the L5 level or higher. Implant survival was 98.9% at five years, with a low revision rate of 0.8%. Complications were rare: 2.7% experienced minor issues and 1.3% experienced major complications. Secondary rotator cuff tears occurred in 1.3% of cases, with only 0.5% requiring revision surgery [4].

#### 4.1.4. Emerging Innovations and Future Directions in ATSA

The increasing use of stemless humeral components is motivated by complications associated with stemmed implants, such as bone loss, malpositioning, periprosthetic fractures, and altered center of rotation [49].

Stemless designs enable three-dimensional reconstruction of the humeral head and its center of rotation independently of the humeral shaft axis, thereby avoiding major osteotomies and their associated complications [49,50,51,52,53]. These implants also offer practical advantages, including reduced surgical time, lower blood loss, and a decreased risk of diaphyseal fractures [54,55]. Two main design types exist, impaction and screw-in systems, both of which have shown comparable loosening rates and functional outcomes [56,57]. Short- to mid-term outcomes (6 months to 5 years) have reported Constant scores ranging from 65 to 86, with revision rates between 0% and 11% [56]. Long-term results (8 to 9 years) show Constant scores between 62 and 69 and revision rates ranging from 7% to 10% [49,58]. Implant-related complications remain rare; however, revisions due to rotator cuff insufficiency have been reported in 13.9% of cases, while periprosthetic joint infections account for 2.3% [49]. Despite encouraging results, stemless implants require adequate bone quality and are not convertible in case of revision, leading to increased surgical complexity and cost [55].

Short-stemmed humeral implants offer several advantages over stemless designs, including a larger surface area for osseointegration and the possibility of conversion in revision settings. Short- to mid-term results show average Constant scores of around 75 and ASES scores near 80, with revision rates reported between 0% and 9% [59,60,61]. Despite some radiographic findings, such as cortical thinning, clinical outcomes remain favorable [60].

Pyrolytic carbon humeral heads, due to their lower modulus of elasticity, may reduce glenoid bone loss and facilitate future revisions; however, data on their use in shoulder arthroplasty remain limited [8,9,62,63].

In cases of significant glenoid retroversion (>15°) or posterior wear, augmented glenoid components offer a promising alternative to standard implants, which are associated with an increased risk of loosening. These augmented components minimize bone removal while enhancing implant stability. Short-term studies report revision rates between 0% and 5%, although higher degrees of augmentation may carry an increased risk of failure [64].

Inlay glenoid components, as opposed to traditional onlay designs, have demonstrated improved biomechanical integration in cadaveric and early clinical studies. These implants may be particularly beneficial in younger, active patients, though long-term data are still lacking [65].

Convertible stem platforms, initially developed to simplify revision from anatomic to reverse arthroplasty, have reduced the need to explant the humeral stem during conversion procedures. These modular systems shorten operative time, decrease intraoperative blood loss, and lower overall revision costs [66]. Studies indicate that in approximately 80% of cases, the initial humeral stem can be preserved during revision surgery, with lower intraoperative complication rates compared to full stem revisions (0% versus 15%) [66,67,68].

More recently, convertible metal-backed glenoid components have emerged as an innovative solution to facilitate conversion from anatomic to reverse shoulder arthroplasty. These components allow for preservation of glenoid fixation and have demonstrated good short- and mid-term outcomes, with revision rates between 0% and 11% and a low rate of complications [69,70,71]. In cases of failed anatomic arthroplasty, their use may help preserve functional gains and simplify the revision process [72].

### 4.2. Reverse Total Shoulder Arthroplasty

**Figure 6:**
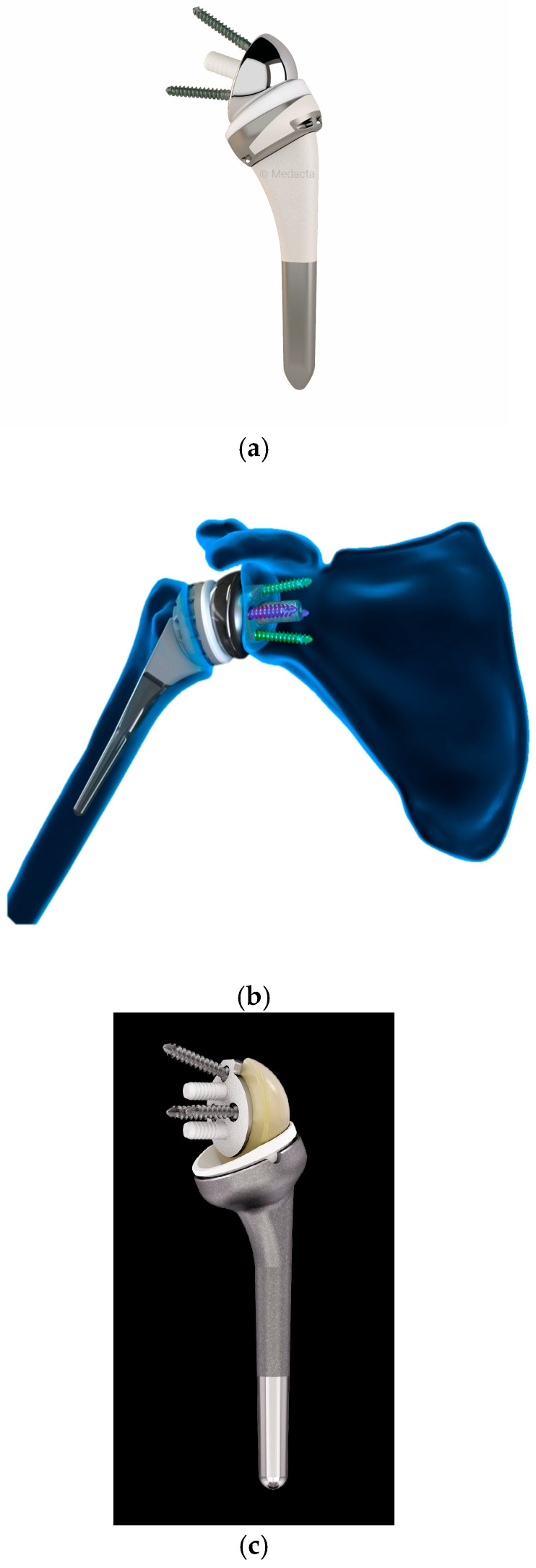
Reverse shoulder prosthesis implant. Pictures of (**a**) Medacta, (**b**) Arthrex, and (**c**) Mathys. The implants shown in this review represent a sample of commonly used systems and do not imply endorsement of a specific manufacturer. Images reproduced with permission from the respective companies.

Reverse total shoulder arthroplasty is primarily indicated in cases of rotator cuff dysfunctions or complex fractures [73,74,75,76,77]. Successful outcomes depend on a functional axillary nerve, full deltoid activity, sufficient bone stock, and normal bone density [78]. RTSA implants feature a medialized and inferiorly positioned center of rotation, allowing the deltoid muscle to compensate for rotator cuff deficiency and generate active shoulder elevation. The concept was introduced by Paul Grammont, whose design offered a reliable solution for shoulders with irreparable or absent rotator cuffs, Figure 6 [79].

While earlier reverse prostheses had limited success, two major design innovations contributed to the effectiveness of the Grammont model: (1) on the glenoid side, a large collarless metallic hemisphere affixed directly to the glenoid surface; (2) on the humeral side, a polyethylene cup with a non-anatomic inclination of 155°, covering less than half the glenosphere [78]. This design medializes and lowers the center of rotation, reducing shear forces on the glenoid, increasing implant stability, and enhancing deltoid efficiency. The resulting relative lowering of the humerus retensions the deltoid muscle fibers, enabling it to restore active anterior elevation [41,78,79].

#### 4.2.1. Range of Motion and Functional Results After RTSA

Although RTSA may result in a slightly reduced range of motion compared to ATSA, it generally provides satisfactory functional outcomes that help preserve quality of life. This has been confirmed by several clinical studies, Table 2.

The role of subscapularis tendon repair in RTSA remains controversial. In a study of 125 patients (mean age: 72 years) with rotator cuff arthropathy, Oak et al. compared outcomes between patients with and without subscapularis repair. Forward elevation was 135° in the repair group and 138° in the non-repair group. External rotation was slightly higher in the non-repair group (30° vs. 27°), while internal rotation was comparable (L3–L4). Patient-reported outcomes—including VAS pain, ASES, and SST scores—showed high satisfaction and no significant differences between the two groups [82].

#### 4.2.2. Revision Rate and Long-Term Survivorship of RTSA

RTSA has demonstrated reliable and reproducible outcomes while providing durable implant survival with a low need for revision, as shown in several clinical studies [73,83,84,85,86]. Nevertheless, potential complications include instability, glenoid loosening, humeral component issues (such as tuberosity resorption, disassembly, or loosening), and scapular notching due to medial impingement between the humeral component and the inferior scapular neck. Recent studies report a revision rate of approximately 10% at 10 years, Table 3 and Table 4.

According to the Australian Shoulder Prosthesis Registry, the revision rate for primary stemmed reverse shoulder arthroplasty is 6.7% at 14 years for primary osteoarthritis and 6.1% at 14 years for rotator cuff arthropathy. Furthermore, a comparison of data collected before 2015 with data from 2015 to 2023 reveals a downward trend in revision rates over time. For the treatment of fractures, the 10-year revision rate for primary stemmed RTSA was reported at 6% [77].

Reverse shoulder arthroplasty is often considered a reliable option for revising failed anatomic prostheses, particularly in cases of glenoid component loosening [88,89,90,91]. However, its outcomes in revision settings remain variable and are associated with significant limitations. Bartels et al. analyzed a cohort of 127 patients who underwent RTSA as a revision procedure for glenoid loosening following anatomic arthroplasty. The patients had a mean age of 71 years and were followed for an average of five years. Postoperative outcomes demonstrated meaningful improvements in shoulder pain and function, with ASES scores increasing from 40 to 67, and a marked reduction in pain levels. Nevertheless, complications occurred in 31% of cases, including infections (9%), instability (7%), and periprosthetic fractures (6%). Moreover, 16% of patients required reoperation, and the five-year implant survival rate was 84%, underscoring the limitations and unpredictability of RTSA in the revision context [92].

#### 4.2.3. Trends in the Use of Reverse Versus Anatomic Total Shoulder Arthroplasty: Clinical Outcomes and Cost Considerations

In recent years, the use of RTSA has increased significantly, particularly among patients presenting with complex pathologies such as irreparable rotator cuff tears or advanced osteoarthritis. This trend has been investigated in several recent studies, which underscore the progressive replacement of ATSA by RTSA in selected clinical scenarios. For instance, Aurich et al. highlighted the expanding use of RTSA in Germany between 2010 and 2022, emphasizing the influence of both the healthcare system and policy changes in supporting this shift [93]. Similarly, Valsamis et al., using data from the National Joint Registry and Hospital Episode Statistics in the United Kingdom, confirmed that RTSA is becoming the preferred treatment option for osteoarthritis in certain subgroups, offering improved outcomes over ATSA and a reduced need for revision procedures [94]. In the United States, Best et al. also reported an increase in the use of RTSA, particularly in patients with irreversible rotator cuff pathology [95].

The 2024 Annual Report from the Australian Orthopaedic Association National Joint Replacement Registry further supports this global trend, indicating that RTSA continues to gain ground, particularly in the management of complex shoulder conditions. Like previous studies, the report emphasizes that RTSA generally exhibits lower revision rates compared to ATSA, especially in patients with severe pathology. However, RTSA may still be associated with complications such as infections or dislocations, which may necessitate more complex revision procedures [77].

From an economic standpoint, Halperin et al. observed that although RTSA is more costly than ATSA, its clinical benefits justify the higher initial expenditure. While cost remains an important factor in treatment decisions, the increasing adoption of RTSA appears to be primarily driven by its clinical advantages, including lower revision rates in patients with complex indications [96].

#### 4.2.4. Emerging Innovations and Future Directions in RTSA

The original Grammont implant featured a 155° inlay design, in which the metaphyseal portion of the humeral stem was inserted into the humeral metaphysis. This configuration increased the contact area and enabled medial positioning of the humerus [78]. Over time, the onlay humeral stem design was introduced, characterized by a metaphyseal tray positioned atop the resected humeral surface. This evolution allowed for greater lateralization and distalization while improving the preservation of proximal bone stock [97,98]. In a biomechanical study conducted by Walch, only the 145° onlay humeral stem demonstrated the capacity to restore more than 50% of the native range of motion while optimizing cuff tension, outperforming both the 155° inlay and the 135°/155° onlay designs [97]. Clinical evaluations have further shown that the onlay configuration yields superior results in adduction, extension, and external rotation compared to traditional inlay stems [70,99]. Although one study reported no significant differences in complication rates, other investigations have noted a higher incidence of scapular fractures associated with distalizing onlay designs [70,98,100].

Stemless RTSA implants offer several advantages, including reduced risk of malposition and periprosthetic fractures, better preservation of proximal bone stock, and easier implantation in cases of altered distal humeral anatomy [101,102]. Several international studies have reported no significant differences in range of motion or clinical outcome scores between stemless and stemmed RTSA implants in short- to mid-term follow-up [103]. However, the presence of osteopenia may increase the risk of early humeral component loosening [53,104].

Managing glenoid bone loss represents a significant challenge in both ATSA and RTSA. In cases of substantial bone loss, RTSA is often preferred due to its constrained design, which helps limit humeral migration and reduces asymmetric polyethylene wear compared to ATSA [105]. Accurate baseplate positioning remains critical and should ideally include 5–10 degrees of neutral version, neutral to slightly inferior inclination, and a minimum of 50% contact with native bone—criteria that can be more readily achieved using augmented baseplates [106]. Augmented glenoids—whether using biological systems (BIO-RTSA) [107] or metallic augmentation [108]—help achieve optimal implant positioning by reducing the need for reaming, preserving native bone, and increasing lateralization [78,109]. Two types of implants are commonly used to address these challenges. Non-custom metallic augmented glenoids, typically available in 10° to 30° wedge configurations, are pre-fabricated and require minimal reaming. In contrast, custom implants are designed based on patient-specific CT imaging and are particularly suited for severe and combined bone loss patterns, especially in revision surgery [110,111,112].

## 5. Conclusions

The evolution of shoulder arthroplasty over recent decades reflects not only the growing number of procedures performed but also significant progress in surgical indications, implant designs, and clinical outcomes. Anatomic total shoulder arthroplasty remains the gold standard for younger, active patients with an intact rotator cuff, although glenoid component loosening continues to be a major concern. Initially developed for irreparable rotator cuff tears, reverse total shoulder arthroplasty has seen a progressive expansion of its indications, driven by consistent outcomes, lower revision rates, and increasingly versatile implant systems. Recent innovations—such as stemless and short-stem components, augmented glenoid designs, and convertible platforms—have enhanced surgical accuracy, facilitated revision procedures, and improved implant longevity. Additionally, pyrocarbon implants offer a promising solution for younger patients by preserving native bone and potentially reducing glenoid wear, although long-term data remain limited. Personalized treatment strategies that consider patient-specific factors—such as age, functional demand, bone quality, and rotator cuff integrity—are essential to achieving optimal outcomes. Continued research and technological advancements will further refine indications and contribute to the long-term success of shoulder arthroplasty.

It is also important to acknowledge that the methodology of analysis and synthesis in this review was inevitably influenced by the availability and limitations of the existing literature. A clear trend has emerged not only in clinical practice but also in scientific publications, where reverse total shoulder arthroplasty has received increasing attention. This focus has led to a notable imbalance in both the volume and specificity of published data. In particular, we encountered considerable difficulty in retrieving studies reporting isolated outcomes for anatomic total shoulder arthroplasty, whereas the literature on reverse implants has grown increasingly abundant and detailed over the past 15 years. This disparity highlights the need for further targeted research on anatomic prostheses, especially in younger and more active patient populations.

## Figures and Tables

**Table 1:** Comparison of clinical outcomes and complications in reverse total shoulder arthroplasty following failed rotator cuff repair.

	Welch et al. (2024) [35]	Tantone et al. (2023) [36]
Study type	Systematic review and meta-analysis	Systematic review and meta-analysis
Population	2149 patients (760 with failed RCR)	1590 patients (527 with failed RCR)
ASES	−8.31 (95% CI: −10.96, −5.66)	−6.12 (95% CI: −8.45, −3.79)
VAS Pain	+0.85 (95% CI: 0.47, 1.22)	+0.58 (95% CI: 0.23, 0.93)
ROM—FF	−6.71° (95% CI: −11.75°, −1.67°)	−9.45° (95% CI: −13.69°, −5.20°)
ROM—ER	No significant difference	−3.61° (95% CI: −5.73°, −1.48°)
Complications	No significant difference	Increased risk (OR = 1.57, 95% CI: 1.12, 2.19)
Revision rates	No significant difference	No significant difference

**Table 2:** Short- and medium-term outcomes of reverse total shoulder prosthesis (RTSA).

Author	Number of Studies	Total Number of Cases	Average Follow-up	Clinical Outcomes
Ernstbrunner et al. [74] 2019	8	365 shoulders	9.5 years	Active forward elevation and abduction showed significant improvement (*p* = 0.004 and *p* = 0.009, respectively). No significant improvement was observed in active external rotation
Bois et al. [80] 2020	43	1041 implants	43.8 months	Except for external rotation, range of motion showed improvement in all groups
Nunes et al. [81] 2021	9	1670 patients	41.1 months	Improvement in forward flexion, abduction, and external rotation

**Table 3:** Revision surgery after reverse shoulder prosthesis (RTSA).

Author	Number of Patients	Revision Rate	Comments
Favard et al. [73] 2011	506 patients (527 prostheses)	Revision rate of 5%	Twelve prostheses were removed due to infection within the first 3 years, with a marked increase in revisions occurring in the first 2 years.
Melis et al. [87] 2011	119 patients (122 prostheses)	Revision rate of 7%	Mainly caused by infections or glenoid loosening.
Bacle et al. [83] 2017	84 patients (87 prostheses)	Revision rate of 12%	Main causes: infection and glenoid loosening.

**Table 4:** Implant survival after reverse shoulder arthroplasty (RTSA).

Author	Results
Favard et al. [73] 2011	89% implant survival at 10 years post-surgery.
Bacle et al. [83] 2017	93% implant survival at 10 years post-surgery.
Goldenberg et al. [85] 2020	Systematic analysis of seven studies on 286 shoulders of patients under 65, showing implant survival rates of 99% at 2 years, 91–98% at 5 years, and 88% at 10 years.
Chelli et al. [84] 2022	91% survival rate after primary RTSA and 80.9% survival rate after revision RTSA. For tumors, survival is 53.1%. In patients under 60, the survival rate is 75.7%, compared to 94.3% in patients over 80.

## Abbreviations

The following abbreviations are used in this manuscript:

NSAID	Non-Steroidal Anti-Inflammatory Drugs
ATSA	Anatomic Total Shoulder Arthroplasty
RTSA	Reverse Total Shoulder Arthroplasty
CT	Computer Tomography
ORIF	Open Reduction Internal Fixation
LPF	Locking Plate Fixation
ASES	American Shoulder And Elbow Surgeons
ROM	Range Of Motion
RCR	Rotator Cuff Repair
VAS	Visual Analog Scale
FF	Forward Flexion
ER	External Rotation
